# Mice with Trp53 and Rb1 deficiency in chondrocytes spontaneously develop chondrosarcoma via overactivation of YAP signaling

**DOI:** 10.1038/s41419-022-04916-4

**Published:** 2022-06-27

**Authors:** Yang Li, Shuting Yang, Yang Liu, Shuying Yang

**Affiliations:** 1 Department of Basic & Translational Sciences, School of Dental Medicine, University of Pennsylvania, Philadelphia, PA 19104 USA; 2 College of Fisheries and Life Science, Dalian Ocean University, 116023 Dalian, China; 3 Center for Innovation & Precision Dentistry, School of Dental Medicine, School of Engineering and Applied Sciences, University of Pennsylvania, Philadelphia, PA 19104 USA; 4 The Penn Center for Musculoskeletal Disorders, School of Medicine, University of Pennsylvania, Philadelphia, PA 19104 USA

**Keywords:** Bone cancer, Bone cancer

## Abstract

Chondrosarcoma (CHS) is a rare type of soft sarcoma with increased production of cartilage matrix arising from soft bone tissues. Currently, surgical resection is the primary clinical treatment for chondrosarcoma due to the poor response to radiotherapy and chemotherapy. However, the therapeutic effect is not satisfactory due to the higher local recurrence rate. Thus, management and elucidation of the pathological mechanism of chondrosarcoma remain an ongoing challenge, and the development of effective chondrosarcoma mouse models and treatment options are urgently needed. Here, we generated a new transgenic chondrosarcoma model by double conditional deletions of Trp53 and Rb1 in chondrocyte lineage which spontaneously caused spinal chondrosarcoma and lung metastasis. Bioinformatic analysis of the human soft sarcoma database showed that Trp53 and Rb1 genes had higher mutations, reaching up to approximately 33.5% and 8.7%, respectively. Additionally, Trp53 and Rb1 signatures were decreased in the human and mouse chondrosarcoma tissues. Mechanistically, we found that YAP expression and activity were significantly increased in mouse Col2-Cre;Trp53^f/f^/Rb1^f/f^ chondrosarcoma tissues compared to the adjacent normal cartilage. Knockdown of YAP in primary chondrosarcoma cells significantly inhibited chondrosarcoma proliferation, invasion, and tumorsphere formation. Chondrocyte lineage ablation of YAP delayed chondrosarcoma progression and lung metastasis in Col2-Cre;Trp53^f/f^/Rb1^f/f^ mice. Moreover, we found that metformin served as a YAP inhibitor, which bound to the activity area of YAP protein, and inhibited chondrosarcoma cell proliferation, migration, invasion, and progression in vitro and significantly suppressed chondrosarcoma formation in vivo. Collectively, this study identifies the inhibition of YAP may be an effective therapeutic strategy for the treatment of chondrosarcoma.

## Introduction

Chondrosarcoma is a rare type of primary bone cartilage malignancy with an incident rate of about two new cases per a million populations per year [1, 2]. It is the second most common primary malignant bone tumor and has a higher local recurrence rate. Although most solid tumors have infrequent metastasis, lung metastasis is the most common in chondrosarcoma [3–5]. It predominantly occurs in adults aged after 40 years old [6]. Currently, surgical resection is the primary clinical treatment for chondrosarcoma due to the poor response to radiotherapy and chemotherapy. However, the therapeutic results are not unfavorable due to the higher local recurrence and mortality rates. Thus, management and elucidation of the pathological mechanism of chondrosarcoma remain an ongoing challenge, and the development of effective chondrosarcoma mouse models and treatment options are urgently needed.

To discover a new drug or for drug repurposing, developing a mouse model that closely mimics human chondrosarcoma initiation and progression is one of the most important approaches in the clinic. Currently, only a few chondrosarcoma animal models have been developed including allograft tumor transplanted into the hamster or rat [7, 8]. Despite these models being more useful for evaluating chondrosarcoma growth, there are less relevant to the human disease that restricts the elucidation of the pathological mechanism and development of novel therapeutic drugs. Transgenic cancer models are becoming more favored because these types of models have been characterized to be accurate models in oncology, which can finely control tumor genetics and more accurately study the tumor initiation and development, and delineate the potential molecular drivers or inhibitors of these pathologies [9, 10]. One transgenic chondrosarcoma mouse model has been developed by transgenically overexpressing c‐Fos. However, the unpredictability of tumor location, varying phenotypes (including osteosarcoma), and multiple tumor formation lead to the potential problem of interpreting data using this model for developing therapies [11, 12].

The identification of some tumor suppressors’ mutations in cancer tissues can provide new strategies and options for the development of the new drug targets. Among these tumor suppressors, the mutations of Trp53 and Rb1 are most well-studied in different tumors. Notably, alterations of Trp53 and Rb1 were observed in about 33-96% and 20-50% of chondrosarcomas, respectively [13–16]. Additionally, the chondrosarcoma originates from cartilaginous tissues that are rich in chondrocytes. However, whether loss of Trp53 and/or Rb1 in chondrocyte lineage can cause chondrosarcoma remains undefined.

Hippo pathway is crucial for skeletal development and tumorigenesis through modulating the activity of the essential downstream effectors Yes-associated protein (YAP) and transcriptional coactivator with PDZ-binding motif (TAZ) [17–21]. The enhanced expression, nuclear localization, and decreased phosphorylation of YAP are frequently observed in various tumors and are also considered novel prognostic markers in sarcoma [20–22]. Of note, although YAP doesn’t have the DNA binding motif, it can function as a key transcription coactivator of other transcriptional factors such as the TEA domain transcription factors (TEADs), Slug, and Snail to regulate cell proliferation and apoptosis [20, 21, 23]. In cancers, hypophosphorylated YAP tends to translocate into the nucleus and further bind with its major partner TEADs, to thereby regulate target genes’ expression [17, 20, 21]. Previous studies showed that YAP expression level is elevated in soft sarcomas including synovial sarcoma and Ewing sarcoma [24, 25] and inhibition of YAP signaling prohibits the lung metastasis potential of Ewing sarcoma cells [25]. In osteosarcoma, we have recently demonstrated that YAP governs the osteosarcoma progression and lung metastasis [17]. Currently, metformin, primarily used for the treatment of type 2 diabetes mellitus, has been reported to effectively inhibit cancers such as uterine cancer, pancreatic cancer, and gastric carcinoma in clinical trials [26, 27]. Moreover, some evidence showed that metformin can decrease YAP expression and increase its phosphorylation level in bladder cancer, and eventually disrupt the formation of YAP/TEAD complex [28]. However, whether metformin can inhibit chondrosarcoma development and lung metastasis is unknown.

Here, we explored the function and molecular mechanisms of Trp53 and Rb1 in driving chondrosarcoma formation and lung metastasis. Our data showed that double deletions of Trp53 and Rb1 in chondrocytes led to the spontaneous development of chondrosarcoma through activation of YAP signaling. Deletion of YAP or inhibition of YAP by metformin significantly inhibited the chondrosarcoma progression in this new Col2-Cre;Trp53^f/f^/Rb1^f/f^ chondrosarcoma mouse model. Thus, this study provides a new transgenic chondrosarcoma model and a proof of principle that the inhibition of YAP activity may be a potential therapeutic target for chondrosarcoma.

## Results

### Trp53 and Rb1 signatures are decreased in human chondrosarcoma

**Fig. 1:**
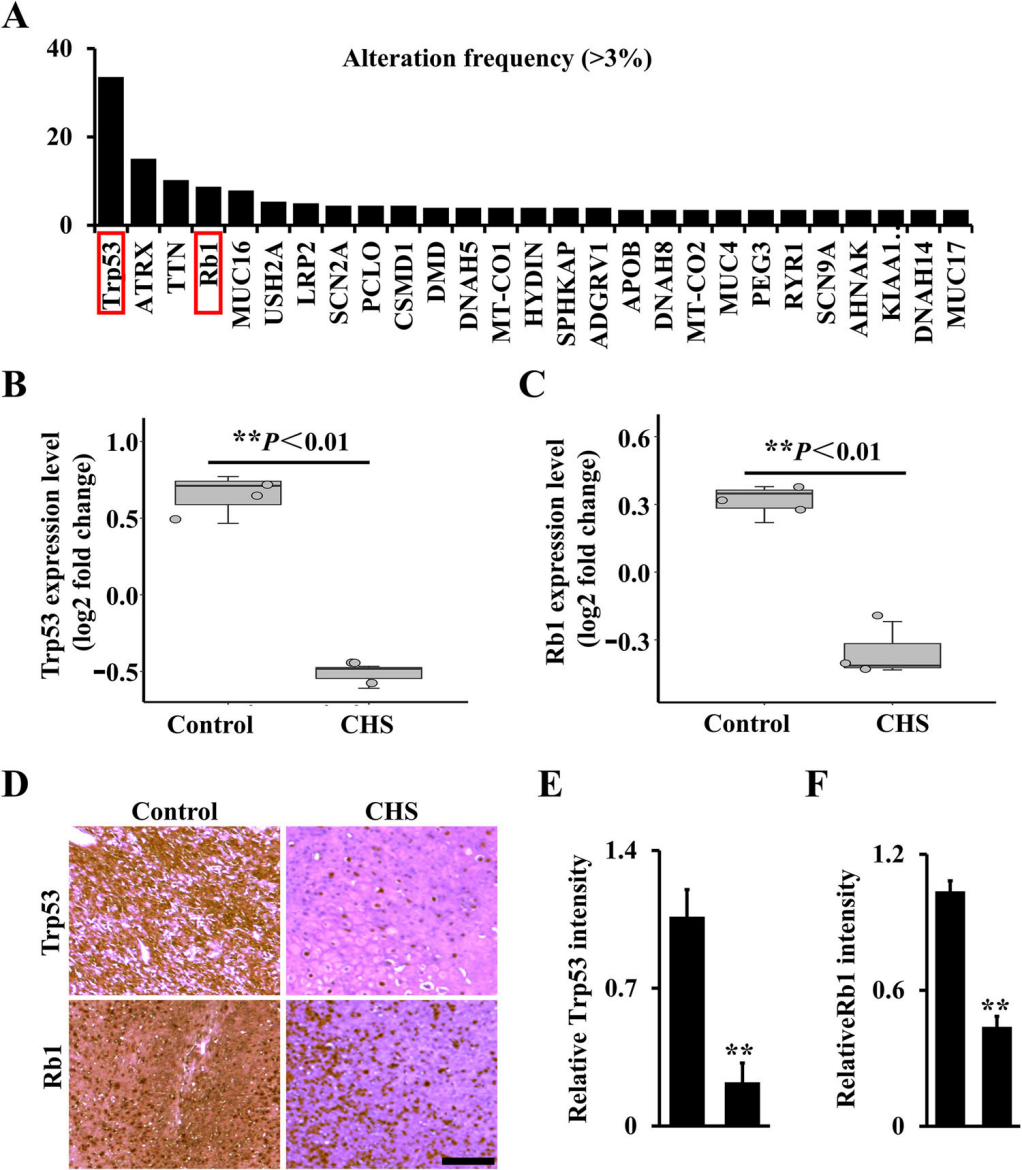
Trp53 and Rb1 signatures are decreased in human chondrosarcoma. **A** The mutant rate of chondrosarcoma >3% from TCGA. The red boxes direct to Trp53 and Rb1. **B**, **C** Trp53 and RB1 expression from GSE48420. **D** IHC analysis of the expressions of Trp53 and Rb1 in human normal cartilage and chondrosarcoma samples as indicated. **E**, **F** The expressions of Trp53 and Rb1 were quantified based on IHC staining by Image J software. *N* = 12. Scale bar, 100 μm. Error bars were the means ± SEM. **P < 0.01.

To understand the function of Trp53 and Rb1 in chondrosarcoma formation, we first analyzed the mutation rates of some tumor suppressors in the soft sarcoma database from TCGA (The Cancer Genome Atlas). Encouragingly, our data showed that Trp53 and Rb1 had higher mutation rates reaching up to 33.5 and 8.7%, respectively (Fig. 1A). Further analysis of Trp53 and Rb1 expressions in human chondrosarcoma cell lines from the dataset available in the GEO database under accession number GSE48420 [29] also showed a significant decrease compared to the normal human chondrocytes (Fig. 1B, C). Consistently, immunohistochemistry (IHC) staining of human chondrosarcoma samples also showed lower expression of Trp53 and Rb1, as evidenced by analysis compared to the normal (Fig. 1D-F).

### Deletion of Trp53 and Rb1 in chondrocytes causes spinal chondrosarcoma and lung metastasis

**Fig. 2:**
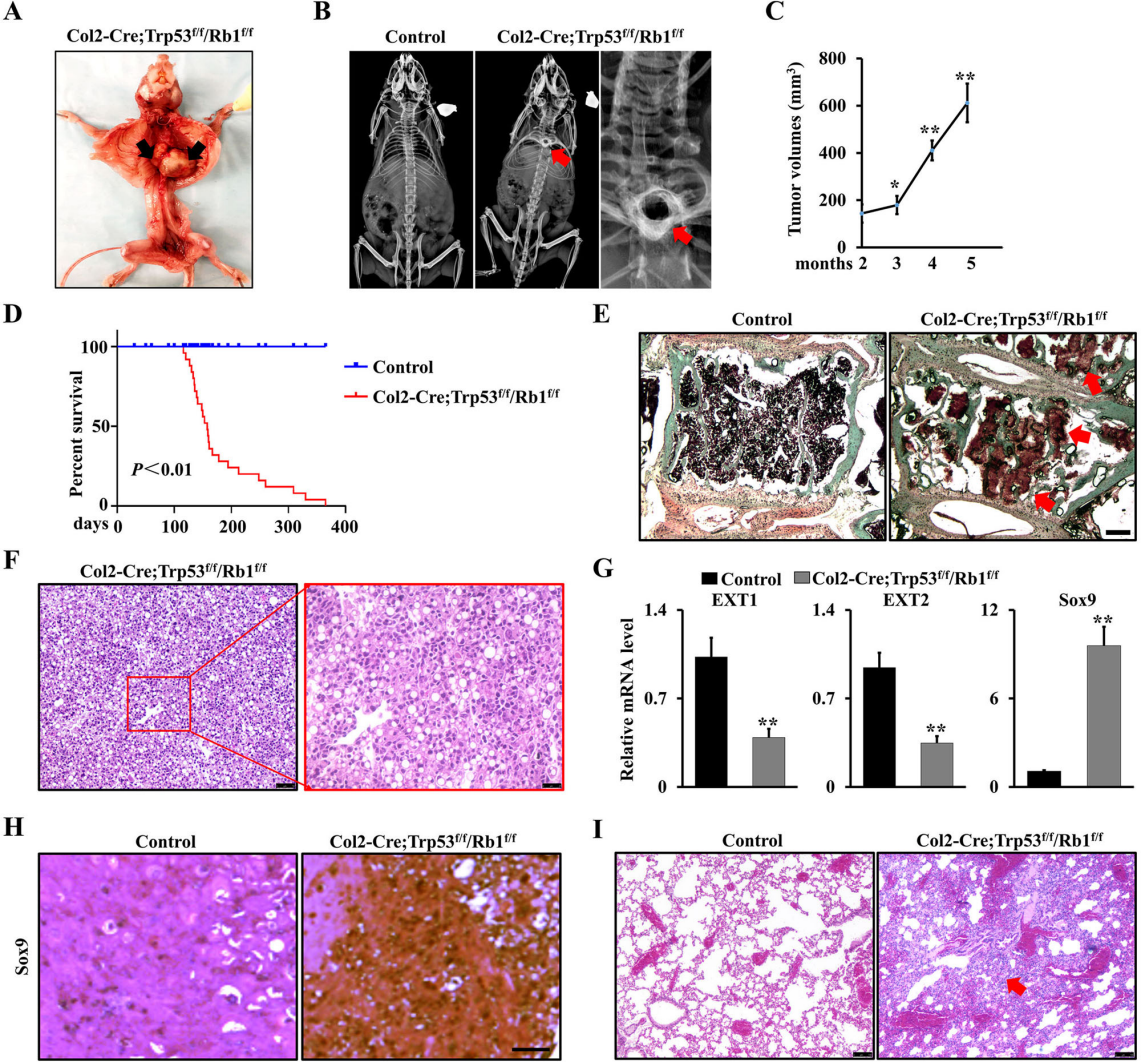
Deletion of Trp53 and Rb1 in chondrocytes causes spinal chondrosarcoma and lung metastasis. **A** Col2-Cre;Trp53^f/f^/Rb1^f/f^ mice at 5 months. **B** X-ray analysis of Col2-Cre;Trp53^f/f^/Rb1^f/f^ mice and controls at age of 5 months. The red arrows direct to the spinal chondrosarcoma. **C** Tumor volume analysis of Col2-Cre;Trp53^f/f^/Rb1^f/f^ mice. **D** Kaplan-Meier survival analysis indicating overall survival of Col2-Cre;Trp53^f/f^/Rb1^f/f^ mice and controls as indicated. *N* = 30. **E** Representative Safranin O/Fast Green staining images of spinal chondrosarcoma from 5-month-old Col2-Cre;Trp53^f/f^/Rb1^f/f^ mice. The red arrows direct to chondrosarcoma in the spine. Scale bar, 75 μm. **F** Representative H&E staining images of chondrosarcoma from 5-month-old Col2-Cre;Trp53^f/f^/Rb1^f/f^ mice. *N* = 3. Scale bar, 100 μm. The high-resolution image was at left as shown. Scale bar, 25 μm. **G** mRNA expression levels of *EXT1*, *EXT2*, and *Sox9* were identified by qRT-PCR in chondrosarcoma from Col2-Cre;Trp53^f/f^/Rb1^f/f^ mice and controls. **H** IHC analysis of Sox9 expression chondrosarcoma from Col2-Cre;Trp53^f/f^/Rb1^f/f^ mice and controls as indicated. *N* = 3. **I** Representative H&E staining images of chondrosarcoma lung metastasis from 5-month-old Col2-Cre;Trp53^f/f^/Rb1^f/f^ mice and controls. *N* = 3. Scale bar, 100 μm. The red arrow directs to chondrosarcoma in the lung. Error bars were the means ± SEM from three independent experiments. *P < 0.05, **P < 0.01.

To further investigate the potential function of Trp53 and Rb1 in chondrosarcoma, we generated the mouse conditional knockout lines in which Trp53 or/and Rb1 were deleted in chondrocyte lineage by crossing Trp53^f/f^, Rb1^f/f^ and Trp53^f/f^/Rb1^f/f^ floxed mice with a transgenic Cre line driven by a Col2a1 promoter (henceforth referred to as Col2-Cre;Trp53^f/f^, Col2-Cre;Rb1^f/f^, and Col2-Cre;Trp53^f/f^/Rb1^f/f^ mice). qRT-PCR data verified that Trp53 and Rb1 were abrogated in chondrocytes (Supplemental Fig. S1A, B). Interestingly, we found single deletion of Trp53 or Rb1 in Col2-expressing cells did not lead to chondrosarcoma formation at observed time points of 1-, 4- and 12-month-old mice (Supplemental Fig. S1C). However, X-ray results showed that Col2-Cre;Trp53^f/f^/Rb1^f/f^ mice exhibited slight bone density changes in the spinal bone at 1 month old, and an apparent disruption in the spinal bone and a big and relatively soft mass surrounding the spinal bone at 4 months, suggesting Col2-Cre;Trp53^f/f^/Rb1^f/f^ mice form spinal chondrosarcoma (Supplemental Fig. S1C). Moreover, Col2-Cre;Trp53^f/f^/Rb1^f/f^ mice at 5 months showed a big mass (Fig. 2A). X-ray image showed a disruption in the vertebrate bone in the thoracic spine region (Fig. 2B). The average volume of chondrosarcoma was increased with age (Fig. 2C). The mice lost walking ability approximately at 4.5 months (Supplemental Video). The Kaplan-Meier survival curves demonstrated a significantly shorter survival rate in the Col2-Cre;Trp53^f/f^/Rb1^f/f^ mice compared to that in the Col2-Cre control mice (Fig. 2D). Moreover, we identified the vertebrate bone architecture of Col2-Cre;Trp53^f/f^/Rb1^f/f^ mice by performing safranin O/fast green staining and found that chondrosarcoma cells were mainly expanded and filled in the marrow cavity of vertebrate bone (Fig. 2E). H&E staining showed intense cellularity and severe cytologic atypia pattern (Fig. 2F). Previous findings suggested that EXT1, EXT2, and Sox9 can be considered genetic markers in the diagnosis and prognosis of chondrosarcoma [30, 31]. In chondrosarcoma tissues, the expression of EXT1 and EXT2 usually has a significant reduction, whereas SOX9 has a higher expression [30, 31]. Consistently, we found the expression of EXT1 and EXT2 significantly decreased, and Sox9 expression increased due to the loss of Trp53 and Rb1 in chondrocytes (Fig. 2G, H). Additionally, we found the chondrosarcoma could metastasize to lungs instead of other organs such as the brain, spleen, kidney, and liver (Fig. 2I and Supplemental Fig. S1D). To confirm the spinal disruption and lung metastasis caused by Trp53 and Rb1 deficiency in chondrocytes directly, we next identified the expression of Trp53 and Rb1 in chondrosarcoma, chondrosarcoma-invaded spine, -metastasized lung tissues and corresponding adjacent normal tissues (Control). As expected, we found that expression of Trp53 and Rb1 was significantly decreased in the chondrosarcoma and chondrosarcoma-invaded tissues compared to controls (Supplemental Fig. S2A–C), suggesting the chondrosarcoma formation and lung metastasis was directly caused by loss of Trp53 and Rb1 in chondrocytes.

### Deletion of Trp53 and Rb1 increases the expansion and differentiation ability of chondrocytes

**Fig. 3:**
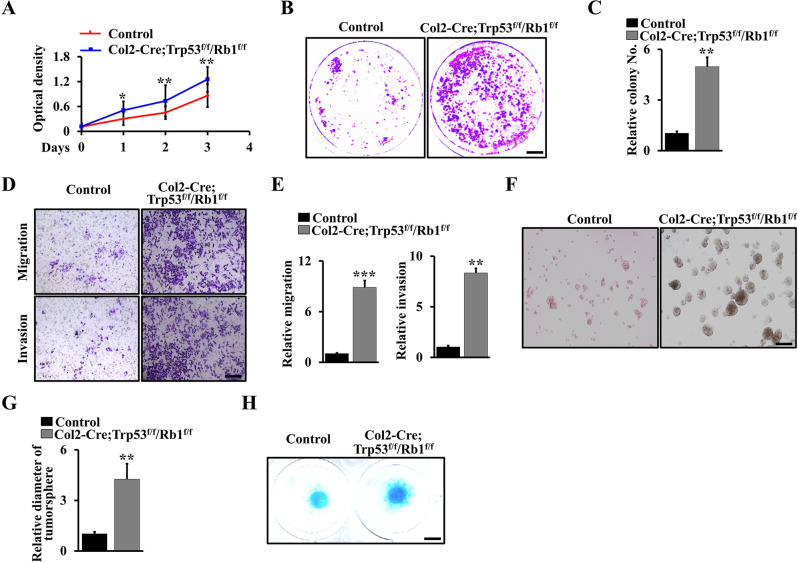
Deletion of Trp53 and Rb1 increases the expansion and differentiation ability of chondrocytes. **A** Cell proliferation rate of primary chondrocytes and chondrosarcoma cells from Col2-Cre;Trp53^f/f^/Rb1^f/f^ mice and age-matched controls (Col2-Cre) as shown by WST-1 assay. **B**, **C** Soft agar after culture of 3 weeks using primary chondrocytes and chondrosarcoma cells from Col2-Cre;Trp53^f/f^/Rb1^f/f^ mice and age-matched controls, respectively (**B**). Cell numbers were quantified in the corresponding column as indicated (**C**). *N* = 3. **D** Migration and invasion as indicated. **E** Migration and invasion were quantified in the corresponding column as indicated. *N* = 3. **F**, **G** Tumorspheres were cultured as indicated for 3 days using primary chondrosarcoma cells from Col2-Cre;Trp53^f/f^/Rb1^f/f^ mice and age-matched controls. The diameter of tumorspheres was quantified in the corresponding column as indicated (**G**). *N* = 3. **H** Micromass culture primary chondrosarcoma cells and chondrocytes from Col2-Cre;Trp53^f/f^/Rb1^f/f^ mice and age-matched controls from Col2-Cre; Trp53^f/f^/Rb1^f/f^ mice and controls. After a culture of 10 days, the macromass cultures as indicated were stained by Alcian blue staining. *N* = 3. Error bars were the means ± SEM from three independent experiments. *P < 0.05, **P < 0.01 ***P < 0.001.

To further explore the mechanism by which Trp53 and Rb1 function in chondrosarcoma formation, we identified the expansion and differentiation ability of chondrocytes after ablation of Trp53 and Rb1 in chondrocytes. We first isolated primary chondrocytes from Col2-Cre;Trp53^f/f^/Rb1^f/f^ mice and controls. Intriguingly, we found the proliferation rate of primary chondrocytes in Col2-Cre;Trp53^f/f^/Rb1^f/f^ mice was significantly increased compared to that in the controls (Fig. 3A). Concomitantly, the colony numbers were also remarkably increased in Trp53 and Rb1 deficient chondrocytes (Fig. 3B, C), suggesting that Trp53 and Rb1 negatively regulated the proliferation and growth of chondrosarcoma cells. The result from the soft agar assay showed an outstanding increase in cell migration and invasion activities after the loss of Trp53 and Rb1 in comparison with controls (Fig. 3D, E). To identify the effect of Trp53 and Rb1 on chondrocyte maturation, we cultured the primary chondrocytes in a chondrogenic differentiation medium. After a culture of 10 days, we found that Trp53 and Rb1 promoted cartilage nodule formation (Fig. 3H).

### YAP signaling acts as a potent driver of the onset and progression of chondrosarcoma

**Fig. 4:**
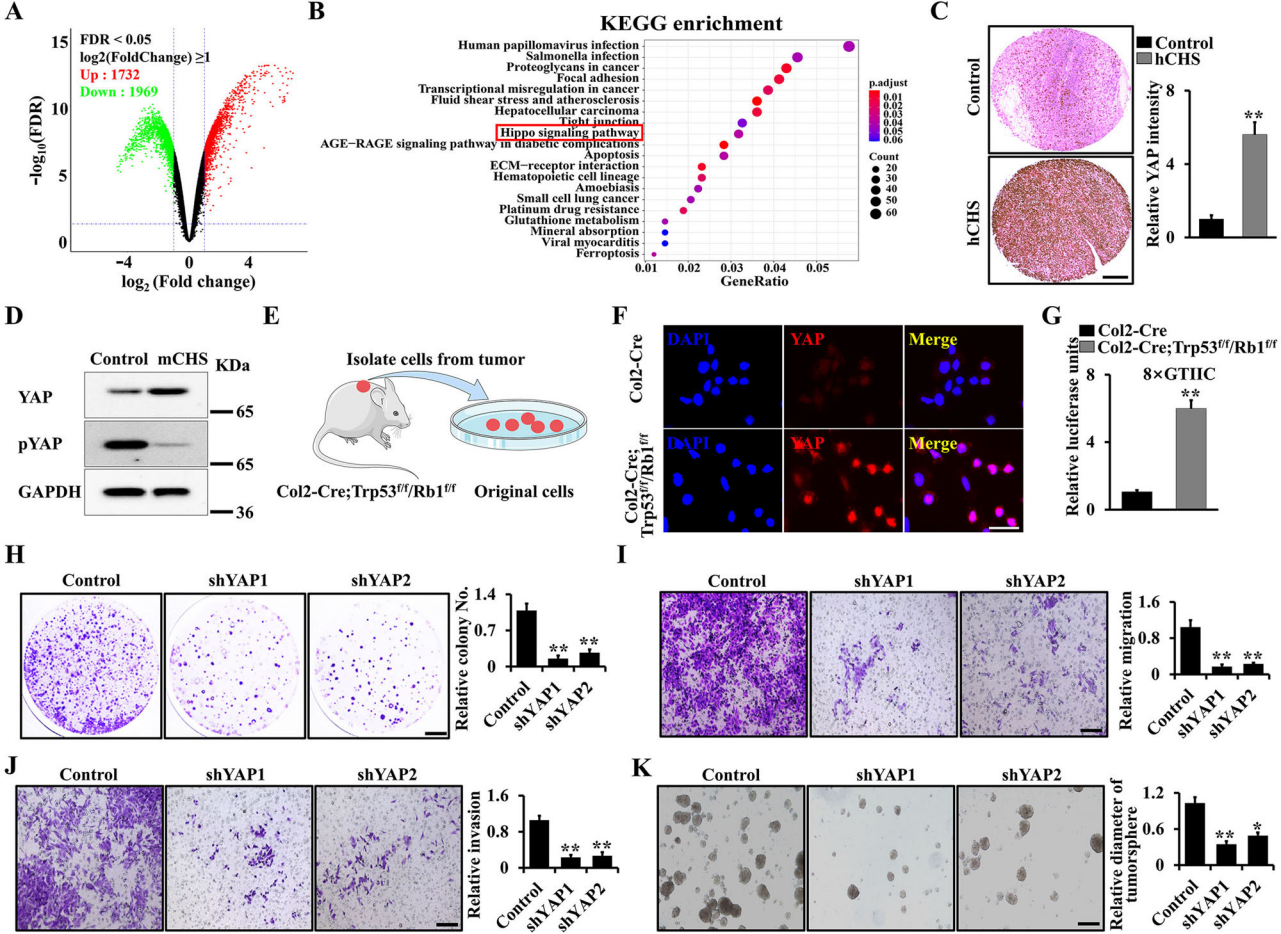
YAP signaling is a potent driver of the onset and progression of chondrosarcoma. **A** Volcano plot of transcriptome profiles between human chondrosarcoma cell lines and controls from GSE48420. **B** KEGG analysis of the chondrosarcoma RNA-seq data showing the top 20 enriched pathways. Red box directs to Hippo pathway. **C** IHC analysis of the expression of YAP in human normal cartilage (control) and chondrosarcoma (hCHS) samples. Quantitative analysis was at right by Image J software. *N* = 12. **D** YAP expression in chondrosarcoma tissues (mCHS) from Col2-Cre;Trp53^f/f^/Rb1^f/f^ mice compared to adjacent normal cartilage (control). **E** Schematic presentation of primary chondrosarcoma cells from Col2-Cre;Trp53^f/f^/Rb1^f/f^ mice. **F** Representative images of immunofluorescent staining of YAP in primary chondrosarcoma cells. Scale bars, 50 μm. **G** The primary chondrosarcoma cells from Col2-Cre;Trp53^f/f^/Rb1^f/f^ mice or primary chondrocytes from Col2-Cre mice were co-transfected with a luciferase reporter and pRL-TK plasmids (internal control) as indicated, respectively. After transfection of 48 h, the luciferase activities were identified by the Dual-Luciferase Assay Kit. **H** Soft agar analysis after silence of YAP using two different YAP lentivirus in primary chondrosarcoma cells from Col2-Cre;Trp53^f/f^/Rb1^f/f^ mice as indicated. The corresponding quantification is identified on the right. *N* = 3. **I**–K The analyses of migration (**I**), invasion (**J**), and tumorsphere (**K**) after silence of YAP in primary chondrosarcoma cells as above. The corresponding quantification is identified on the right. *N* = 3. Error bars were the means ± SEM from three independent experiments. *P < 0.05, **P < 0.01.

To further define the mechanism by which Trp53/Rb1 regulates chondrosarcoma progression, we analyzed publicly available human chondrosarcoma data from GSE48420 [29]. Volcano plot of those database showed a set of significantly downregulated (1969) and upregulated (1732) genes with more than 2-fold change compared to normal chondrocytes (Fig. 4A). Among these genes, the Hippo pathway exhibited a conserved signature as one of top significantly enriched gene sets (Fig. 4B). Moreover, YAP expression level increased in both human chondrosarcoma tissues (Fig. 4C) and Col2-Cre;Trp53^f/f^/Rb1^f/f^ mouse chondrosarcoma tissues compared to adjacent normal cartilage (Fig. 4D). Given single gene deletion of Trp53 and Rb1 didn’t cause chondrosarcoma formation, however, the joint deletion led to the occurrence of chondrosarcoma, these results indicated that these two genes may have a mutual compensation effect on the regulation of YAP. Next, we identified the expression of YAP and its target genes CYR61 and CTGF in Trp53 or/and Rb-mutant chondrocytes. As expected, we found that double deletions of Trp53 and Rb1 could result in a significantly increased expression and transcriptional activity of YAP compared to that in the single deletion of Trp53 or Rb1 (Supplemental Fig. S3A, B). Immunofluorescence assay also revealed an enhanced endogenous YAP nuclear localization in primary chondrosarcoma cells from Col2-Cre;Trp53^f/f^/Rb1^f/f^ mice compared to the controls (Fig. 4E, F). To further examine YAP/TEAD1 transcriptional activity, we conducted 8xGTIIC-luciferase assay which was confirmed with high specificity and sensitivity [17, 32, 33]. The result showed a significantly increased transcriptional activity of YAP in Trp53/Rb1-deficient chondrocytes (Fig. 4G). Given that YAP activity was increased in chondrosarcoma, we next silenced YAP in primary chondrosarcoma cells from Col2-Cre;Trp53^f/f^/Rb1^f/f^ mice using two different YAP shRNA lentivirus (Supplemental Fig. S4) to identify whether inhibition of YAP can suppress chondrosarcoma progression. Our data showed that knockdown of YAP significantly inhibited anchorage-independent cell growth in soft agar (Fig. 4H). and reduced ability of cell migration and invasion compared with the controls (Fig. 4I, J). Additionally, the result from tumorsphere culture showed that knockdown of YAP remarkably decreased the formation and diameter of tumorspheres (Fig. 4K). Hence, these results indicated that YAP is a potent driver of chondrosarcoma.

### Metformin inhibits chondrosarcoma progression through YAP signaling

**Fig. 5:**
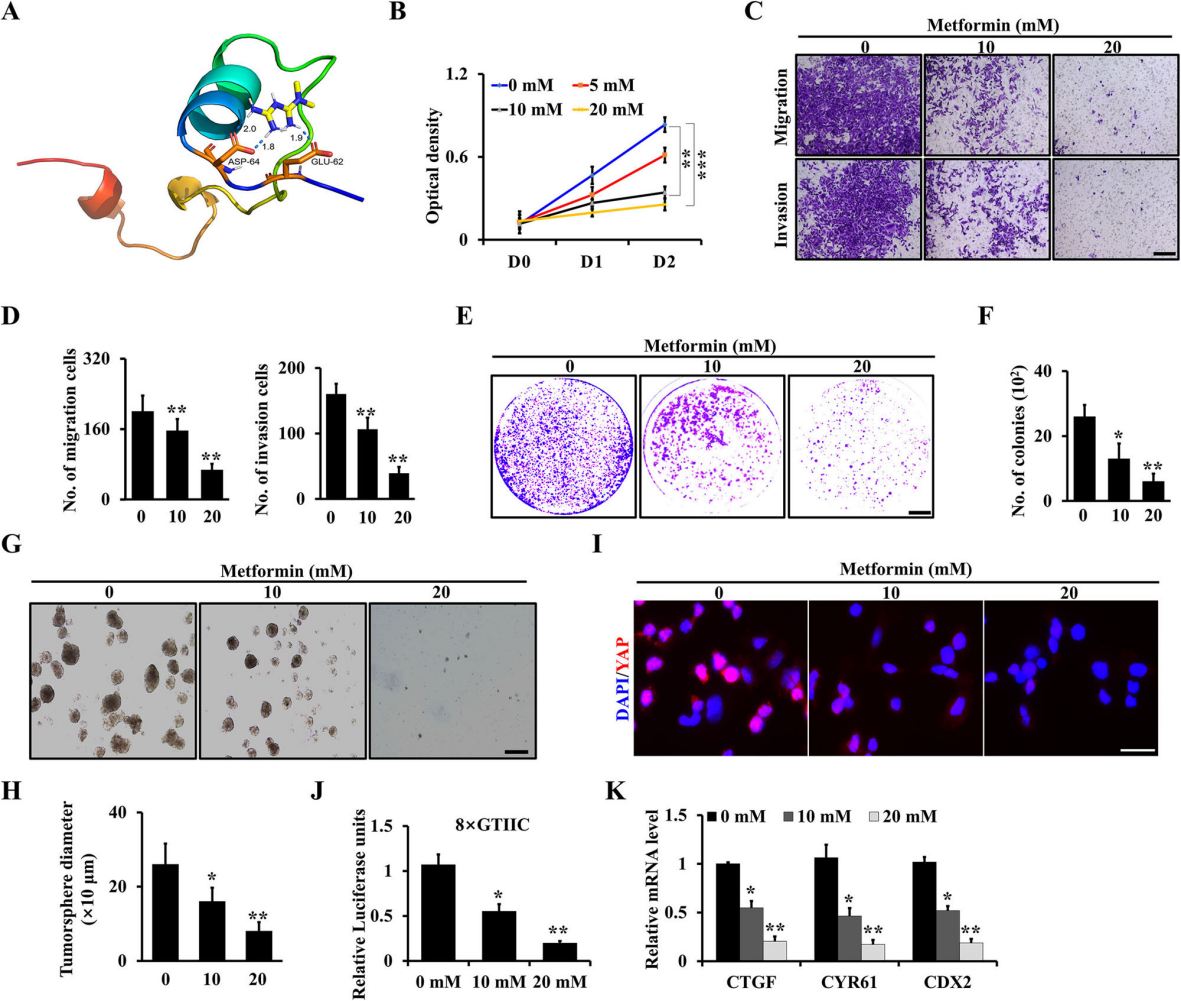
Metformin inhibits chondrosarcoma progression through YAP signaling. **A** Docking between YAP (6GE3 from PDB) and metformin. **B** After treatment of different doses of metformin as shown, the cell proliferation was determined at D0, D1, and D2 using primary chondrosarcoma cells from Col2-Cre;Trp53^f/f^/Rb1^f/f^ mice. **C** Representative images of migration and invasion after treatment of different doses of metformin as indicated using primary chondrosarcoma cells. **D** The quantitative analysis of migration and invasion based on **C**. *N* = 3. **E**, **F** Representative images of soft agar (**E**) and quantitative analysis (**F**) after treatment of metformin as shown for 3 weeks. *N* = 3. **G**, **H** Representative images of tumorsphere (**G**) and quantitative analysis (**H**) after treatment of metformin for 3 days as indicated. *N* = 3. **I** Metformin inhibits YAP nuclear translocation. **J** After treatment with metformin of 48 h, luciferase activities were identified as indicated. **K** qRT-PCR analysis of YAP target genes’ expression. Error bars were the means ± SEM from three independent experiments. *P < 0.05, **P < 0.01.

Emerging evidence indicates that metformin regulates the formation of YAP/TEAD complex [26, 28]. To test the role of metformin in regulating YAP activation in chondrosarcoma, we performed a molecular docking between YAP and metformin. Interestingly, we found that metformin could bind to the activity area [34] (6GE3 from PDB) of YAP protein and prohibit the interaction of YAP and TEAD (Fig. 5A). To further characterize the effects of metformin on chondrosarcoma progression, we explored WST-1 assay to examine the effect of metformin on chondrosarcoma cell proliferation. As expected, we found metformin prohibited the chondrosarcoma cell growth in a dose-dependent manner (Fig. 5B). Moreover, the activities of migration and invasion of primary chondrosarcoma cells were significantly inhibited after treatment with metformin compared with the controls (Fig. 5C, D). Metformin inhibited anchorage-independent chondrosarcoma cell colony formation in soft agar (Fig. 5E, F) and the formation and size of tumorspheres, respectively (Fig. 5G, H). Mechanistically, we found that metformin inhibited the nuclear localization and transcriptional activity of YAP (Fig. 5I, J), and significantly reduced YAP target genes’ expression (Fig. 5K). Collectively, our findings suggested that metformin inhibits chondrosarcoma progression through YAP signaling.

### Inhibition of YAP significantly suppresses chondrosarcoma progression in Col2-Cre;Trp53^f/f^/Rb1^f/f^ mice

**Fig. 6:**
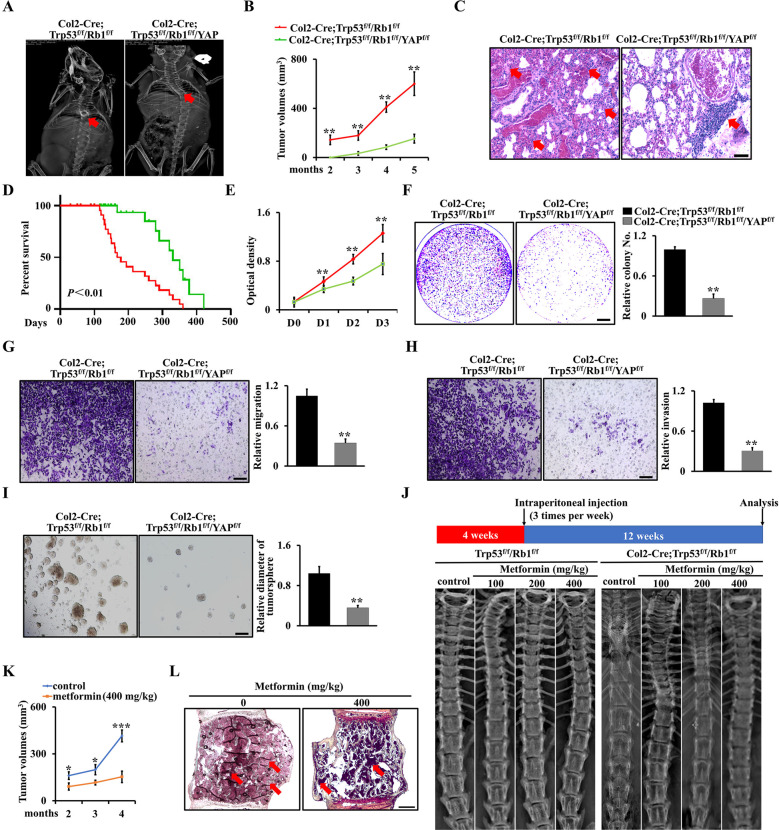
Inhibition of YAP signaling delays chondrosarcoma progression in Col2-Cre;Trp53^f/f^/Rb1^f/f^ mice. **A** Representative X-ray images of Col2-Cre;Trp53^f/f^/Rb1^f/f^ and Col2-Cre;Trp53^f/f^/Rb1^f/f^/YAP^f/f^ mice at 6 months. The red arrows direct to the destruction in the spine. **B** Tumor volume analysis as indicated. *N* = 5. **C** Representative H&E staining images of chondrosarcoma lung metastasis as indicated. *N* = 3. Scale bar, 100 μm. **D** Kaplan–Meier survival analysis indicating overall survival of Col2-Cre;Trp53^f/f^/Rb1^f/f^ mice and controls as indicated. *N* = 30. **E** Cell proliferation rate of chondrosarcoma cells from Col2-Cre;Trp53^f/f^/Rb1^f/f^ and Col2-Cre;Trp53^f/f^/Rb1^f/f^/YAP^f/f^ mice as shown by WST-1 assay after D0, D1, D2, and D3. **F**–**I** Representative images and quantitative analysis of soft agar (**F**), migration (**G**), invasion (**H**), and tumorsphere (**I**) using primary chondrosarcoma cells from Col2-Cre;Trp53^f/f^/Rb1^f/f^ and Col2-Cre;Trp53^f/f^/Rb1^f/f^/YAP^f/f^ mice as indicated. *N* = 3. **J** Representative X-ray images of spines after treatment with metformin (100, 200, and 400 mg/kg) or PBS (control) three times per week for 3 months as indicated. *N* = 5. **K** Tumor volume analysis after treatment with 400 mg/kg metformin or PBS (control) three times per week for 3 months as indicated. *N* = 5. **L** Representative Safranin O/Fast Green staining images of spinal chondrosarcoma from Col2-Cre;Trp53^f/f^/Rb1^f/f^ mice after treatment with metformin (0 and 400 mg/kg) three times per week for 3 months as indicated. *N* = 3. The red arrows direct to chondrosarcoma in the spine. Error bars were the means ± SEM from three independent experiments. *P < 0.05, **P < 0.01, ***P < 0.001.

To corroborate the above observations and assess the role of YAP on the chondrosarcoma progression in Col2-Cre;Trp53^f/f^/Rb1^f/f^ mice (double cKO mice), we constructed a triple conditional knockout mouse model Col2-Cre;Trp53^f/f^/Rb1^f/f^/YAP^f/f^ (triple cKO mice). As expected, our X-ray data showed that deletion of YAP could partly protect against the spinal bone destruction and display a decreased volume of chondrosarcoma in Col2-Cre;Trp53^f/f^/Rb1^f/f^ mice, suggesting that inactivation of YAP in Col2-Cre;Trp53^f/f^/Rb1^f/f^ mice could block chondrosarcoma progression (Fig. 6A, B). Furthermore, we also found that chondrosarcoma lung metastasis was inhibited by loss of YAP (Fig. 6C). Additionally, the Kaplan-Meier survival curves plotted for the mice showed a significantly longer mean survival rate in the triple cKO mice compared with the double cKO mice (Fig. 6D). To test the capacity to promote tumor growth after the loss of YAP, we performed WST-1 and soft agar assays using chondrosarcoma cells from triple cKO mice and double cKO mice. Noteworthy, the tumor growth was significantly inhibited after the loss of YAP (Fig. 6E, F). Meanwhile, deletion of YAP significantly inhibited cell migration and invasion (Fig. 6G, H). In accordance with the reduced mobility of osteosarcoma cells, loss of YAP remarkably decreased the numbers and size of tumorsphere compared with that in the double cKO group (Fig. 6I). To investigate the role of metformin in vivo, we further used metformin to treat the Col2-Cre;Trp53^f/f^/Rb1^f/f^ mice via intraperitoneal injection three times per week starting at 4 weeks of age, when the spine began to expand in Col2-Cre;Trp53^f/f^/Rb1^f/f^ mice (Fig. 6J). The analysis of X-ray images and chondrosarcoma volume indicated that metformin significantly inhibited tumor growth and improved the mobility of Col2-Cre;Trp53^f/f^/Rb1^f/f^ mice at the age of 4 months (Fig. 6J, K), as evidenced by Safranin O/Fast Green staining (Fig. 6L).

## Discussion

Chondrosarcoma is a rare type of soft sarcoma with increased production of cartilage matrix arising from soft bone tissues [35, 36]. If no effective therapeutic options are taken that would lead to a below approximately 30% survival rate in the following 10 years in the patients with unresectable or metastatic chondrosarcoma. Previous findings showed that there are four different kinds of chondrosarcoma including mesenchymal chondrosarcoma, dedifferentiated chondrosarcoma, clear cell chondrosarcomas, and extraskeletal myxoid [31]. It is well known that mesenchymal chondrosarcoma is a highly aggressive malignant tumor and has a high risk of distant metastasis [31]. Our results showed that Trp53 and Rb1 deficiency in chondrocytes caused chondrosarcoma with serious disruption in the vertebrate bone and lung metastasis. Moreover, Sox9 was reported to be a useful marker in distinguishing mesenchymal chondrosarcoma from other primitive small cell malignancies [30]. Intriguingly, our qPCR and IHC data showed a significant increase in Sox9 expression, indicating that Trp53 and Rb1 deficiency in chondrocytes likely results in a subtype of chondrosarcoma-mesenchymal chondrosarcoma formation. Overall, we first demonstrated that loss of Trp53 and Rb1 in chondrocytes caused spinal chondrosarcoma and lung metastasis, and the inhibition of YAP expression and activity may be therapeutically valuable.

Mutation of Trp53 and Rb1 has been found in many kinds of human tumors especially sarcoma [37–39]. In consistent with that, by performing a bioinformatic analysis of the human soft sarcoma database from TCGA, we found the mutant rates of Trp53 and Rb1 reached approximately 33.5 and 8.7%, respectively. Intriguingly, we found double conditional deletions of Trp53/Rb1 in chondrocytes using Col2-Cre resulted in spinal chondrosarcoma formation and lung metastasis, but single deletion of Trp53 or Rb1 didn’t cause chondrosarcoma. The incidence of spinal chondrosarcomas in humans is about 2–12%, and the thoracic spine is the most frequent localization, followed by the cervical and lumbar region [40]. Almost all patients have symptoms of pain and a palpable mass and bout 50% of patients also have neurologic symptoms. Similar to these symptoms, we found Col2-Cre;Trp53^f/f^/Rb1^f/f^ mice lost walking ability due to the pain and disruption of the spine, vertebrate bone, and neurologic deficits. Chondrosarcoma is the second most common primary malignant bone tumor after osteosarcoma. Previous studies showed that single deletion of Rb1 in mesenchymal cells and osteoblasts could not cause osteosarcoma formation [38, 41]. However, it’s well-known that single deletion of Trp53 alone in osteoblast precursors using OSX-Cre develops spontaneous osteosarcoma and survives for approximately 10 months [41]. Chondrocytes usually function as earlier than osteoblasts. Unexpectedly, we didn’t find spinal chondrosarcoma formation after deletion of Trp53 alone in Col2-Cre and the mice can be survived at age of 12 months. It’s possible that Trp53 has not yet caused chondrosarcoma formation, but chondrogenesis has stopped. Because chondrocytes-mediated chondrogenesis generally is considered to play critical functions at the earliest phase during skeletal development and then gradually becomes weaker. Indeed, how the Trp53 and RB1 precisely coordinate the function of osteoblast and chondrocyte lineages is largely unknown, which needs to be further verified in the future. We believe that our first findings will develop and validate a new pre-clinical chondrosarcoma mouse model and test a new therapeutic strategy for combating chondrosarcoma.

YAP as a core regulator of the Hippo pathway is crucial for tumorigenesis and skeletal development by regulating cell proliferation, differentiation, and apoptosis, and is also considered as a prognostic biomarker in many tumors [19, 21, 42]. Our previous study showed that YAP has an elevated expression and directs the osteosarcoma progression and lung metastasis [17]. Trp53 deficiency cooperates with elevated expression of YAP to promote tumorigenesis with an altered differentiation of original cells [43, 44]. Inactivation of Trp53, or combined loss of Trp53 and Rb1, in mammary epithelium, has been approved to result in mammary carcinomas that bear recurrent YAP amplifications [43]. What’s more, these carcinomas appear to become more sensitive and addicted to YAP overexpression. In consistent with that, we found the Hippo-YAP pathway is a conserved signature as one of the top significantly enriched gene sets in soft sarcoma, and the YAP signature was increased in both human and mouse chondrosarcoma tissues compared to adjacent normal cartilage. More importantly, we found loss of Trp53 and Rb1 in chondrocytes promoted YAP expression and nuclear translocation and elevated the YAP/TEAD1 transcriptional activity. On the contrary, knockdown of YAP showed a reduced ability of chondrosarcoma cell migration and invasion and tumor formation. In vivo, we found loss-of-function of YAP delayed chondrosarcoma progression and lung metastasis in Col2-Cre;Trp53^f/f^/Rb1^f/f^ mice and the Kaplan–Meier survival curves plotted for the mice showed a significantly longer mean survival rate in the triple cKO mice compared with the double cKO mice. All these findings suggested that Hippo-YAP signaling may be a potent driver of the onset and progression of chondrosarcoma.

Metformin is the first-line drug for the treatment of type 2 diabetes [27]. Recent studies demonstrated metformin exhibits strong anti-tumor functions in some tumor cell and mouse models [45, 46]. Additionally, there is strong evidence for the interplay between decreased tumor incidence and metformin treatment [47, 48]. The current studies also highlight the fact that metformin could inhibit the transcriptional activity and expression of YAP. Consistently, our data showed that metformin could bind to the activity area of YAP protein directly and prohibit its transcriptional activity and chondrosarcoma progression, indicating that metformin is a potential new drug for the treatment of chondrosarcoma. Supportively, other studies also demonstrated that metformin inhibits tumorigenesis through disruption of YAP/TEAD complex formation and inhibition of YAP-mediated target genes’ transcription [28, 42]. In vivo, inhibition of YAP signaling by metformin significantly suppressed chondrosarcoma formation. Collectively, this study provides a new transgenic chondrosarcoma model and identifies the inhibition of YAP may be an effective therapeutic strategy for the treatment of chondrosarcoma.

## Materials and methods

### Animals and human samples

Col2-Cre, and YAP^f/f^ mice were purchased from The Jackson Laboratory (Bar Harbor, USA). Trp53^f/f^/Rb1^f/f^ mice were a gift from Dr. David M. Feldser’s lab at the Department of Cancer Biology, University of Pennsylvania. The human chondrosarcoma and normal cartilage samples were purchased from the US Biomax company (#T261b, BO2081; USA).

### Antibodies and reagents

Trp53 (1C12), YAP (D8H1X), GAPDH antibodies, and Hippo Signaling Antibody Sampler Kit were purchased from Cell Signaling Technology. Antibody against Rb1 was purchased from Santa Cruz Biotechnology. The secondary fluorescent antibodies and H&E staining kit were from Abcam. DAB Substrate Kit was ordered from Vector Laboratories Inc. Plasmids pRL-TK, 8xGTIIC-luciferase, and shYAP1/2 were obtained from Addgene. The transfection reagents (FuGENE^®^ HD) were obtained from Promega Corporation.

### Cell culture and micromass

Primary chondrocytes in this study were isolated from the embryonic limb buds of Col2-Cre;Trp53^f/f^/Rb1^f/f^ mice and controls, respectively. Primary chondrosarcoma cells were isolated from chondrosarcoma of Col2-Cre;Trp53^f/f^/Rb1^f/f^ mice. Briefly, the fresh embryonic limb buds and chondrosarcoma were cleaned after removing all around soft tissues and cut into pieces and then dissociated with Trypsin solution (Fisher Scientific™, USA) at 37 °C for 30 min. Subsequently, these cells after digestion were harvested and cultured in α-MEM (Gibco, USA) supplemented with 10% FBS (Gibco, USA) and 1% Pen-Strep solution (Gibco, USA) at 37 °C with 5% humidified CO_2_, and the medium was replaced every other day. Micromass cultures were performed as previously described [18].

### qRT-PCR

Briefly, 1 μg total RNA was extracted from primary chondrocytes of Col2-Cre, Col2-Cre;Trp53^f/f^, Col2-Cre;Rb1^f/f^, and Col2-Cre;Trp53^f/f^/Rb1^f/f^ mice, or primary chondrosarcoma cells from Col2-Cre;Trp53^f/f^/Rb1^f/f^ mice using TRIzol reagent (TaKaRa, Japan) was reverse-transcribed into cDNA by PCR using PrimeScript™ RT Kit (TaKaRa, Japan). qRT-PCR was then performed by CFX96 Real-Time PCR System and the SYBR Green mixture (Bio-Rad, USA). GAPDH was served as an internal control and was determined by 2^–∆∆Ct^ method. The primers of qRT-PCR in this study were listed in Supplemental Table S1.

### Cell functional assays

Cell proliferation, migration, invasion, and tumorsphere assays were carried out as we performed previously [17, 49].

### Luciferase reporter assay

For luciferase reporter assay, the chondrocytes were seeded and co-transfected with luciferase reporter and the indicated plasmids in the 12-well plate. After culturing for 48 hrs, the luciferase activities were analyzed by the Dual-Luciferase Assay Kit as we performed previously [17, 49].

### Radiographic procedures analysis

Radiographic procedures were performed in the Siemens X-ray equipment (Madison, WI, USA) as we performed previously [17, 49–51].

### Histological analysis

Vertebrate bones from 5-month-old Col2-Cre;Trp53^f/f^/Rb1^f/f^ mice and controls were harvested, fixed in 4% paraformaldehyde (PFA) overnight at 4 °C, decalcified with 14% EDTA in PBS (pH 7.4) for 1 month, and then embedded in paraffin. Soft tissues including the lung, kidney, spleen, brain, and liver from Col2-Cre;Trp53^f/f^/Rb1^f/f^ mice and controls were fixed and embedded in paraffin. Six-micrometer sections of the above tissues were prepared, and then the stainings of Haemotoxylin and Eosin (H&E), Safranin O/fast green, and Alcian blue staining were conducted as we previously reported [17, 18, 50].

### Immunofluorescence and immunohistochemistry

For immunofluorescence staining, the primary chondrocytes from 5-month-old Col2-Cre;Trp53^f/f^/Rb1^f/f^ and Col2-Cre mice were seeded and cultured on coverslips. After a culture of 48 h, the coverslips were fixed in 4% PFA for 5 min at room temperature and then permeabilized with 0.3% Triton X-100 in PBS (PBST) 3 times per 5 min each. Next, the cells were blocked by 1% BSA for 1 hr at room temperature and probed with primary antibody against rabbit anti-YAP (1:200 dilution) overnight at 4 °C. After washing 3 times with PBST, the cells were incubated with Alexa Fluor^®^ 594-conjugated second anti-rabbit antibody (1:1000 dilution) for 1 hr at dark. Then, a counterstain of nuclei was performed with DAPI and washed 3 times with PBST, and then the cells were visualized under a fluorescence microscope as we previously reported [17, 18, 50]. Immunohistochemistry was carried out as we previously reported [17, 50].

### Western blot

Briefly, the fresh chondrosarcoma tissues and adjacent normal cartilage from Col2-Cre;Trp53^f/f^/Rb1^f/f^ mice were harvested and lysed with RIPA lysis buffer and protein inhibitor cocktail (Fisher Scientific™, USA), respectively. And then, an equal amount of the above proteins were subjected to SDS-PAGE gel (Bio-Rad, USA), transferred to a PVDF membrane (GVS Life Sciences, USA), and immunoblotted with primary antibodies YAP, pYAP, and GAPDH (1:1000 dilution) for overnight at 4 °C. Following 3 times washing with 0.1% TBST (Tween-20 in TBS), the PVDF membranes were incubated with HRP-conjugated anti-rabbit antibody (1:1000 dilution) for 1 hr at room temperature. After washing 3 times with TBST, the membranes were analyzed with ECL solution (Thermo Fisher, USA) as we previously reported [17, 18, 50].

### Tumor volume analysis

After euthanasia of the mice at indicated different time points, the width and length of chondrosarcomas from Col2-Cre;Trp53^f/f^/Rb1^f/f^, Col2-Cre;Trp53^f/f^/Rb1^f/f^/YAP^f/f^, and Col2-Cre;Trp53^f/f^/Rb1^f/f^ with the treatment of 400 mg/kg metformin mice and controls were measured using vernier calipers. And then, the chondrosarcoma volumes were calculated with the formula length × width^2^ × 0.5 [52, 53].

### Docking and bioinformatic analysis

The structures of YAP protein (6GE3) and metformin were obtained from the Protein Data Bank (PDB) and PubChem database of the National Center for Biotechnology Information (NCBI). The YAP-metformin docking was performed using PyMOL and AutoDock software. Public available human chondrosarcoma cells and normal chondrocytes database from Gene Expression Omnibus (GEO, ID: GSE48420) [29] were used to determine the genes’ fold change and KEGG analysis. For each gene, significant differences in expression were identified using the two-tailed, Student’s t-test, with *p* values <0.05 considered statistically. All data were downloaded and analyzed by R packages DESeq2 and ClusterPfofiler.

### Statistical analysis

Experimental results were reported as mean ± SEM and analyzed by the software of SPSS 21, and the data were analyzed using Student’s *t* test. The statistical significance of multiple groups was determined by a two-way analysis of variance. *P* values <0.05 were considered significant.

## Supplementary information

SUPPLEMENTAL MATERIAL

video

checklist

## Data Availability

All data needed to evaluate the conclusions in the paper are present in the paper and/or the Supplementary Materials.
