# Supplementary material for: Mice with Trp53 and Rb1 deficiency in chondrocytes spontaneously develop chondrosarcoma via overactivation of YAP signaling
Source: Cell Death Dis. 2022 Jun 27;13(6):570. doi: 10.1038/s41419-022-04916-4 (PMC9237030; doi:10.1038/s41419-022-04916-4)
Supplement: Supplementary file 1 — SUPPLEMENTAL MATERIAL [file 41419_2022_4916_MOESM1_ESM.docx]

**Supplemental Figures and Legends**

**Fig. S1** **Deletion of Trp53 and Rb1 in chondrocytes causes chondrosarcoma and lung metastasis.** **A**, **B** mRNA expression levels of *Trp53* and *Rb1* were identified by qRT-PCR as indicated. **C** Representative X-ray images of Col2-Cre;Trp53^f/f^, Col2-Cre;Rb1^f/f^, Col2-Cre;Trp53^f/f^/Rb1^f/f^ mice and controls as indicated timepoint. Single deletion of Trp53 and Rb1 didn’t cause chondrosarcoma formation as indicated timepoint. N=5. **D** Representative H&E staining images of brain, spleen, kidney, and liver in 6-month-old Col2-Cre;Trp53^f/f^/Rb1^f/f^ mice and controls (Col2-Cre). N=5. The red arrow directs to chondrosarcoma. Scale bar, 100 μm. Error bars were the means ± SEM from three independent experiments. **P* < 0.05, ***P* < 0.01, ****P* < 0.001.

**Fig. S2 The expression of Trp53 and Rb1 in different chondrosarcoma tissues.** **A-C** Trp53 and Rb1 expression in chondrosarcoma, chondrosarcoma-burdened spine and lung tissues compared to corresponding adjacent normal tissues (control) as indicated.

**Fig. S3 YAP expression and activity. A** YAP expression in chondrocytes from Col2-Cre;Trp53^f/f^, Col2-Cre;Rb1^f/f^, Col2-Cre;Trp53^f/f^/Rb1^f/f^ mice and controls as indicated. **B** YAP target genes *CTGF* and *CYR61* were identified by qRT-PCR as indicated. Error bars were the means ± SEM from three independent experiments. ***P* < 0.01, ****P* < 0.001.

**Fig. S4 YAP mRNA level after silence of YAP using two different lentivirus in primary chondrosarcoma cells from Col2-Cre;Trp53^f/f^/Rb1^f/f^ mice.** Error bars were the means ± SEM from three independent experiments. **P* < 0.05.

**Fig. S1**


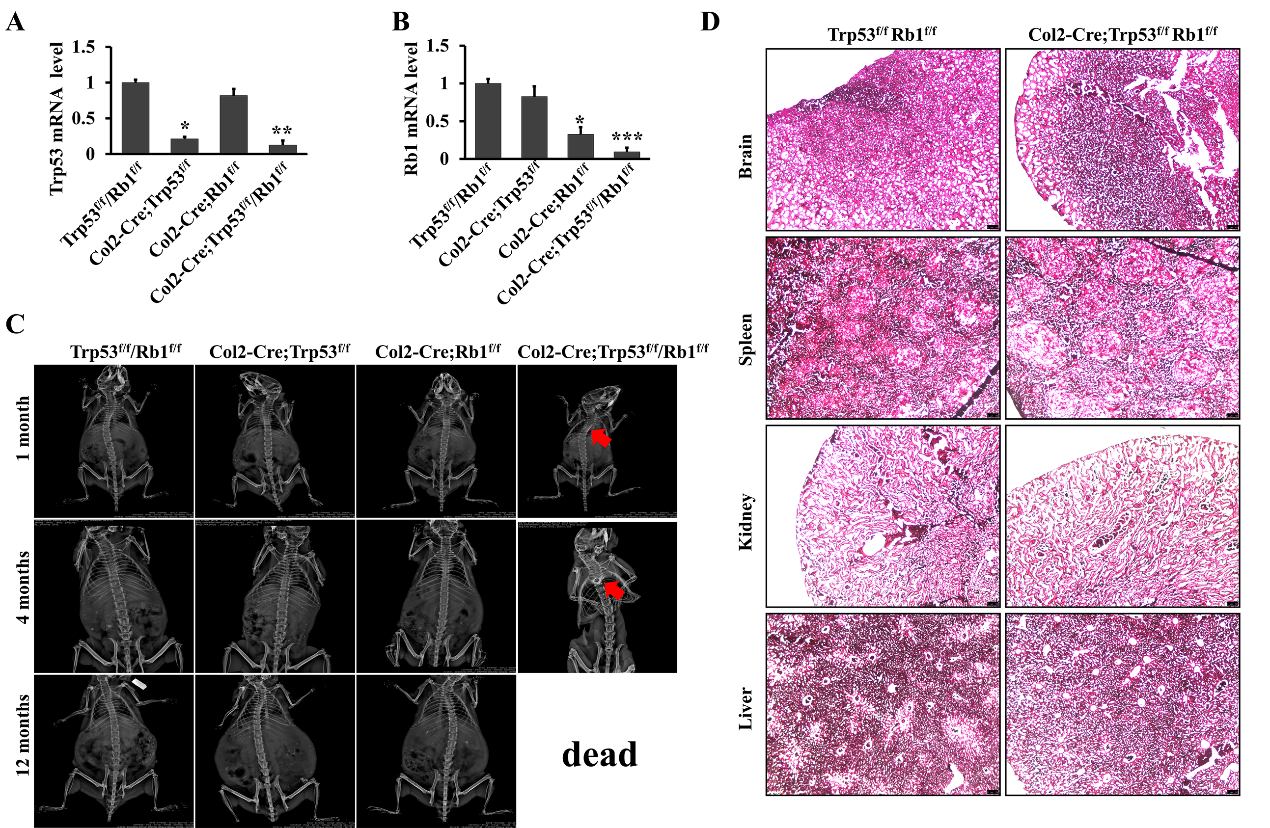


**Fig. S2**


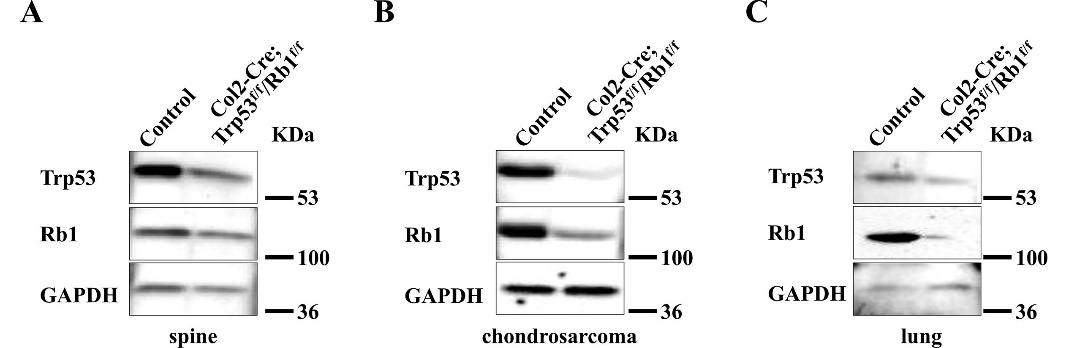


**Fig. S3**


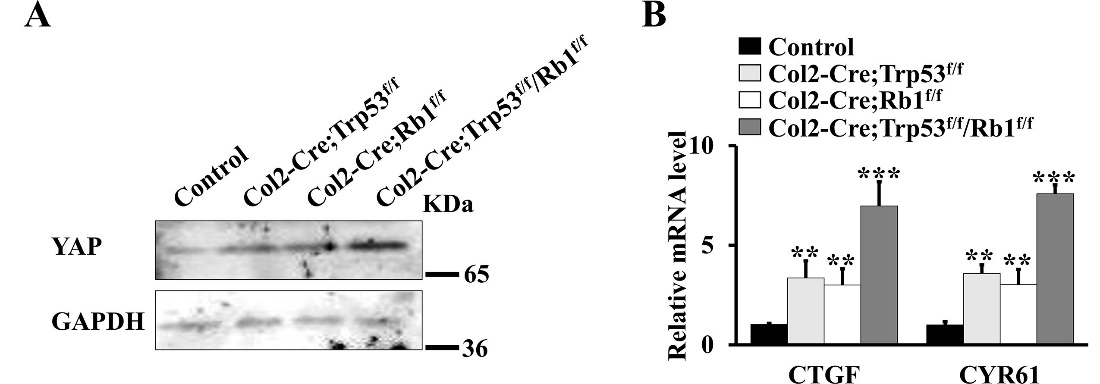


**Fig. S4**


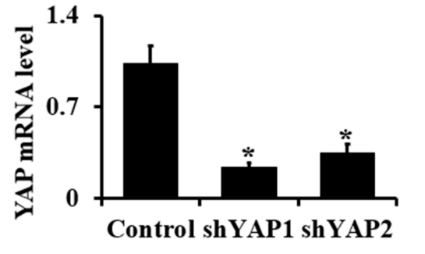


**Supplementary information, Table S1**

| **Gene** | **sequence （5'-3'）** |
| --- | --- |
| GAPDH-F | CCTGGTCACCAGGGCTGCCATTT |
| GAPDH-R | CGTTGAATTTGCCGTGAGTGGAG |
| YAP-F | GGGGACTCGGAGACCGACTTGGA |
| YAP-R | AGGAGTCGGGCAGCTTGCGAAGC |
| Trp53-F | ACTATGGCTTCCACCTGGGC |
| Trp53-R | GCTGGCAGAATAGCTTATTGAGGG |
| Rb1-F | TGGGAGAAAGTTTCATCCGTGG |
| Rb1-R | GCATCTCATCTAGATCAACTGCTGCG |
| Cyr61-F | GCCCTCTGGAGGCACCCAAGTGC |
| Cyr61-R | GGTCGCAGGGCTGAGTTTTGCTG |
| CTGF-F | GCAGTGCACACTCCGATCTTGCG |
| CTGF-R | CCTGGTCACCAGGGCTGCCATTT |
| CDX2-F | CCTGAACCTGGCTCCGCAGAACTT |
| CDX2-R | TGTCCAAGTTCGCCGTAGCAGC |
| EXT1-F | GAAAACGAGGATTCCAGCGT |
| EXT1-R | CTCCATGCGGCACTTCTTGC |
| EXT2-F | AGAGCGGGGGGATCTCAGCTGTAGA |
| EXT2-R | CGGAACACCGGCATCATCCA |
| Sox9-F | CCAGGAGAACACCTTCCCCA |
| Sox9-R | TCGTAGCCCTTCAGCACCTG |
